# CytoSorb haemoadsorption in critically ill children: a multicentre observational study on feasibility, safety, and outcomes, the CYTOPED study

**DOI:** 10.1016/j.aicoj.2026.100073

**Published:** 2026-04-30

**Authors:** Gabriella Bottari, Stefania Bianzina, Anna Tessari, Monica Fae, Sara Giovannini, Stefano Scollo, Elena Caporossi, Fabio Paglialonga, Pietro Lonardi, Raffaella Sagredini, Alessandro Simonini, Fabio Caramelli, Germana Longo, Corrado Cecchetti, Andrea Moscatelli, Isabella Guzzo

**Affiliations:** a Pediatric Intensive Care Unit, Children Hospital Bambino Gesù, IRCSS, Rome, Italy; b Neonatal and Pediatric Intensive Care Unit, Department of Emergency Medicine, Anesthesia, and Critical Care, IRCSS, Istituto Giannina Gaslini, Genova, Italy; c Pediatric Intensive Care Unit, Department of Woman's and Child's Health, University of Padova, Padova, Italy; d University Hospital of Bologna, General and Pediatric Anesthesia and Intensive Care Department, Bologna, Italy; e Cardio-Thoracic and Vascular Anesthesia and Intensive Care Unit, IRCCS Azienda Ospedaliero-Universitaria di Bologna, Italy; f Depatment of Critical Care Medicine, The Hospital for Sick Children, Toronto, Canada; g San Vincenzo Hospital, Mediterranean Pediatric Cardiac Center, Taormina, Italy; h Department of Cardiothoracic and Vascular Intensive Care, University Hospital Integrated Trust of Verona, Italy; i Pediatric Nephrology and Dialysis Unit, University of Milan, Italy; j Pediatric Nephrology Unit Regina Margherita Children's Hospital Torino, Italy; k Children Hospital Burlo Garofalo, Pediatric Intensive Care Unit, Trieste, Italy; l Pediatric Anesthesia and Intensive Care Unit, Salesi Children’s Hospital, Ancona, Italy; m Dialysis Unit, Bambino Gesù Children Hospital, IRCSS, Rome, Italy

**Keywords:** Hemadsorption, Paediatric critical care, Extra-corporeal therapies, Septic shock, Liver failure, Rhabdomyolysis

## Abstract

**Introduction:**

CytoSorb® is a cartridge for the adsorption of inflammatory mediators, bilirubin, myoglobin and other xenobiotics, directly from the blood stream. Clinical experience is widely documented in adults, whereas, in the paediatric settings, it is currently limited to single case reports or monocentric studies. In order to be able to collect evidence in larger paediatric populations, an Italian multicentre network (CYTOPED study group) was founded.

**Methods:**

Italian multicentric observational registry on the use of CytoSorb® in critically ill paediatric patients. Prospective enrolment by Italian Children’s Hospitals has been ongoing since February 2021 with a retrospective analysis conducted from February 2018 to February 2021.

**Results:**

62 patients have been enrolled. Median Paediatric Logistic Organ Dysfunction 2 (PELOD-2) score on Paediatric Intensive Care Unit (PICU) admission was 7 (IQR 4;10). The primary clinical indications for haemoadsorption (HA) were sepsis or septic shock (*n* = 36), followed by liver failure, rhabdomyolysis, cardiac surgery. CytoSorb® has been applied in 87% of cases integrated in a continuous renal replacement therapy (CRRT) circuit. The median time of HA was 48 h (IQR 26;72) and the median number of cartridges used was 2 (IQR 1;3). Anticoagulation in the extracorporeal circuit has been managed with heparin (76%) and regional citrate anticoagulation (24%). Adverse events were recorded in 12 patients.

**Conclusion:**

Our data provide some insights into safety and feasibility of CytoSorb® therapy in children. The advancement of the study and the prospective arm of CYTOPED registry will allow further investigation into this therapy, including dosage, timing and use of antibiotics in conjunction with extracorporeal blood purification techniques.

## Graphical abstract

**Figure:**
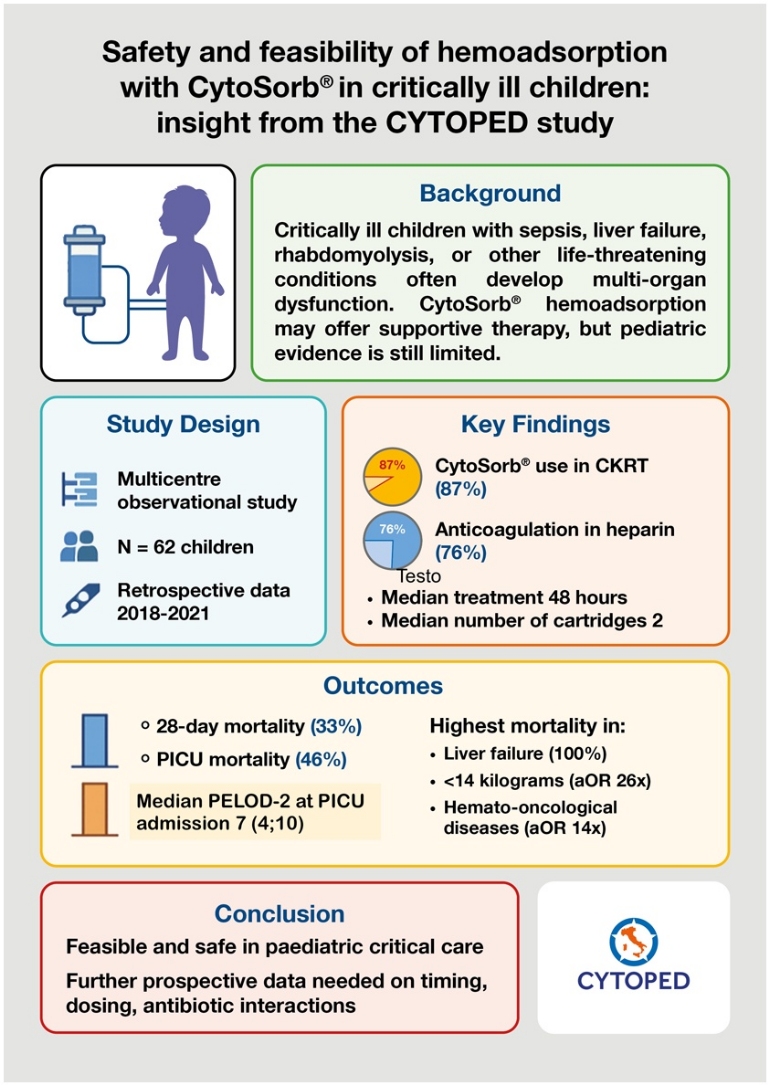

## Introduction

Adsorption is an extracorporeal blood purification technique that aims to remove mediators, solutes, cells, or pathogens through plasma or haemoadsorption (HA) [1]. HA presents several advantages making the diffusion of these techniques exponentially increasing in the last 10 year. Compared to conventional dialysis techniques, absorption is able to enhance the removal of target mediators such as endotoxins, cytokines, and myoglobin: in contrast respect to plasma separation, it is more specific and focused on medium molecular weight mediators [1].

One of the most investigated devices is CytoSorb®, a polystyrene-divinylbenzene resin with the ability to adsorb target molecules with a molecular weight up to 60 kDa, including inflammatory mediators, bilirubin, myoglobin and other xenobiotics, directly from whole blood [2]. The clinical experience of CytoSorb® in different clinical settings has been widely documented in adults [3,4], whereas in paediatrics, it is currently limited to single case reports or monocentric studies [5,6]. However some studies have recently reported encouraging results in the paediatric population, highlighting potentially greater benefits compared to the adult population [7]. In order to be able to collect evidence in larger paediatric populations an Italian multicentre network was founded in 2021: the CYTOPED study group.

The aim of the current analysis is to report treated patients’ characteristics and treatment data with a particular focus on technical considerations and overall outcomes of critically ill children who were supported with CytoSorb® in the paediatric intensive care unit (PICU). To the best of our knowledge, this is the first study designed to demonstrate the feasibility and safety of CytoSorb® in a multicentre paediatric cohort across different clinical indications.

## Material and methods

### Population characteristics

The study was approved by the research ethics committee (REC) of the Children’s Hospital, Bambino Gesù [Ethical approval n°136 (07/2023)]. Informed consent was obtained from the parents or the child’s next of kin. All study procedures complied with the ethical standards and guidelines of the Research Ethics Committee (REC) for human experimentation and adhered to the principles of the 1975 Declaration of Helsinki and following amendemnts. Each participating centre followed its own institutional protocols regarding the timing, criteria, and prescription for initiating and terminating Cytosorb® HA therapy.

### Study design and outcomes

The CYTOPED registry is an observational, retrospective and prospective multicentre, national data collection electronic platform. Here is reported the retrospective section of the registry, including data collection on critically ill children treated with CytoSorb® HA between February 2018 and February 2021 in 12 Italian centres.

This retrospective observational analysis assessed the treatment feasibility in real-world clinical practice, reporting the treatment administration, duration, and tolerability including PICU and 28 day survival. Furthermore the analysis reported a description of adverse events, thereby providing information on treatment’s safety.

### Data collection

Data collection on admission (enrolment) included demographics, comorbidities, complex high-intensity therapeutic strategies, primary and secondary diagnoses, the Paediatric Logistic Organ Dysfunction 2 score (PELOD-2), which allows assessment of the severity of cases of multiple organ dysfunction syndrome in the PICU with a continuous scale [8], the vasoactive inotropic score (VIS) [9]. The CYTOPED collection electronic platform includes different sections dedicated to different HA clinical indications including sepsis, liver failure, treatment during cardiac surgery, rhabdomyolysis, and other minor indications (including cytokine storm syndromes, microangiopathies, intoxications). Treatment information recorded included details on the delivery of HA with CytoSorb and extra-corporeal circuits, device flow rate, and anticoagulation regimen. PICU and 28- day mortality, and adverse events details were collected systematically in the registry. Adverse events collected included: severe hypotension, defined according to age-adjusted mean arterial pressure values and occurring within one hour of CytoSorb connection to the circuit; severe hemodilution, defined as hemoglobin < 7 g/dL in the absence of active bleeding during HA treatment; and thrombocytopenia, defined as a platelet count variation greater than 20% compared with baseline during HA. Furthermore, a free-text field to report other types of adverse events was included in the database.

### Statistical analysis

The current analysis aimed to gather data of CytoSorb® use in critically ill children. A descriptive analysis and data summary was performed to reveal patient characteristics and outcomes without a formal hypothesis testing. For the continuous variables the median and interquartile range (IQR) were reported and for categorical variables, counts and proportions were provided. To identify potential factors associated with patient 28-day survival, logistic regression analyses were performed. Given the relatively small sample size and the potential imbalance in outcome distribution, Firth’s penalized maximum likelihood estimation was applied to reduce small-sample bias and improve the stability of the estimates.

We considered *p* < 0.05 to be statistically significant. All statistical analyses were performed using the R software (version 3.3.3) or SAS software version 9.4 (Cary, NC, USA).

## Results

### Patient demographic data

**Fig. 1:**
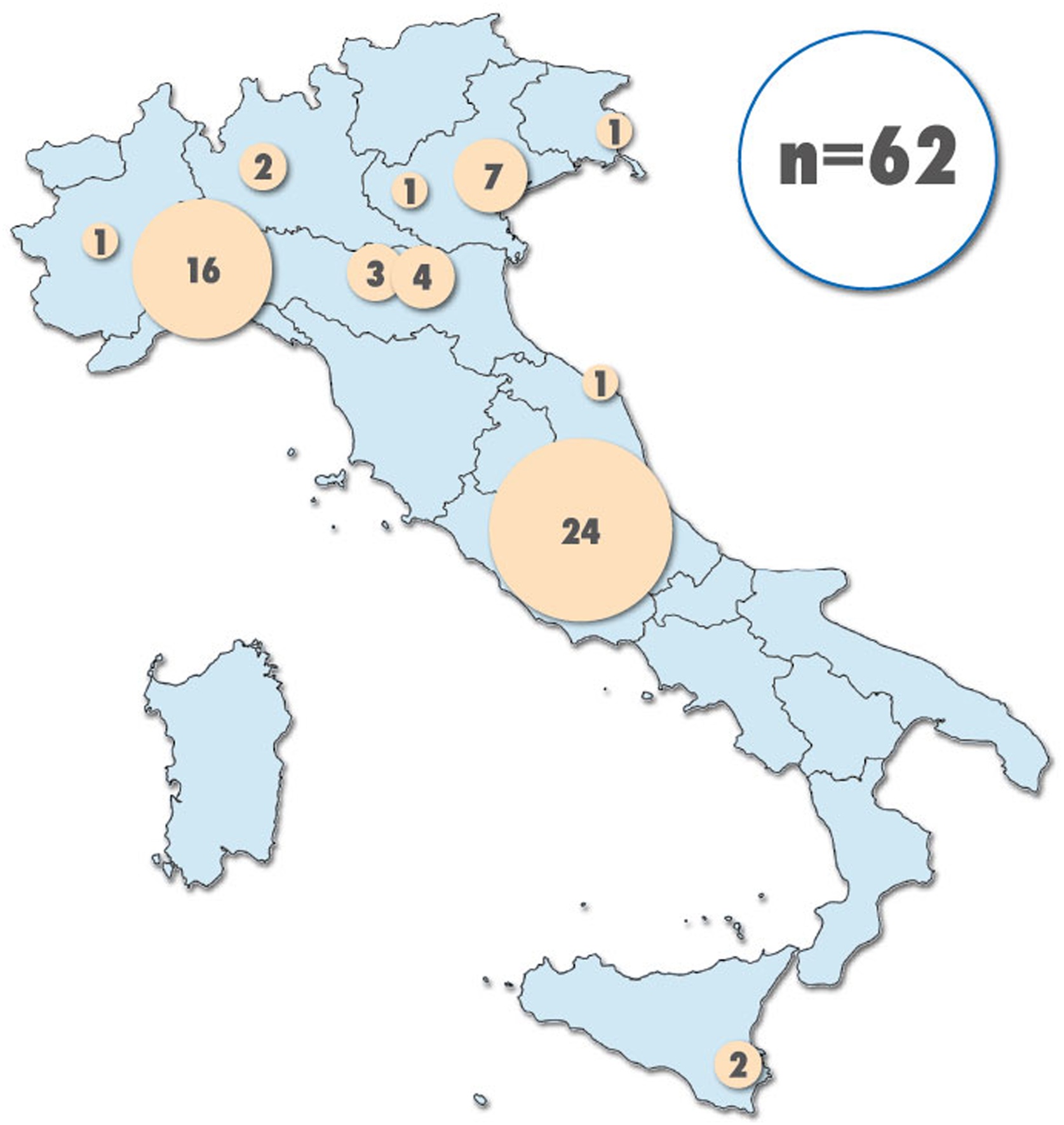
The figure illustrates the distribution of patients recruitment across the Italian Paediatric Intensive Care Unit within the Italian Cytoped network.

Sixty-two children (25 male) received treatments with CytoSorb®. Fig. 1 represents the patient recruitment rate among Italian paediatric hospitals. Table 1 summarizes the characteristics of the population at PICU admission. The majority of patients (79%) was admitted to the PICU due to a medical indication, while 10% was admitted for a surgical related diagnosis.

**Table 1:** Characteristics of the population at PICU admission.

Characteristics of the population at PICU admission	
Number of patients	62
% of females	37
Median Age (months) (IQR)	66 (12; 165)
Median weight (Kg) (IQR)	20.5 (11; 40)
Median PELOD-2 (IQR)	7 (4; 10)
Median PELOD-2 mortality (%)	3.5 (0.9; 13)

**Comorbidities (%)**	**74%**
Complex congenital heart defects	9
Non-cardiac congenital malformations	12
Heart failure	9
Chronic respiratory failure	5
Chronic pulmonary hypertension	6
Liver failure	5
Chronic kidney failure	6
Hematological malignancies	12
Solid tumors	3
Bone marrow transplant	3

**Complex high-intensity therapeutic strategies (%)**	**52%**
Immunosuppressive drugs	15
High-dose corticosteroid therapy	8
Chronic hemodialysis	2
Immunoglobulins	5
Anticoagulants	9
Advanced immunotherapies (immunotherapies including monoclonal antibodies and CAR-T cells)	9

### Modality of CytoSorb delivery across extra-corporeal systems

A total of 137 treatments were administered in the analysed paediatric population. The median number of cartridges used was 2 (IQR 1;3), and the median duration of treatment was 48 h (IQR 26;72). Among the various extracorporeal modalities, 54 patients (87%) received HA with CytoSorb® in combination with Continuous Renal Replacement Therapy (CRRT), 4 patients underwent HA as a stand-alone therapy, 3 patients during cardiopulmonary bypass (CBP), 4 patients received CytoSorb® during extra-corporeal membrane oxygenation (ECMO). Of the ECMO cases, three were combined with both CRRT and ECMO support. Anticoagulation during CRRT was achieved with systemic heparin infusion in 76% of cases and regional citrate anticoagulation in 24%. Among CRRT recipients, the most frequent modality was continuous veno-venous hemodiafiltration – CVVHDF – (89%), followed by continuous veno-venous hemodialysis – CVVHD – (5.5%) and continuous veno-venous hemofiltration – CVVH – (5.5%).

**Fig. 2:**
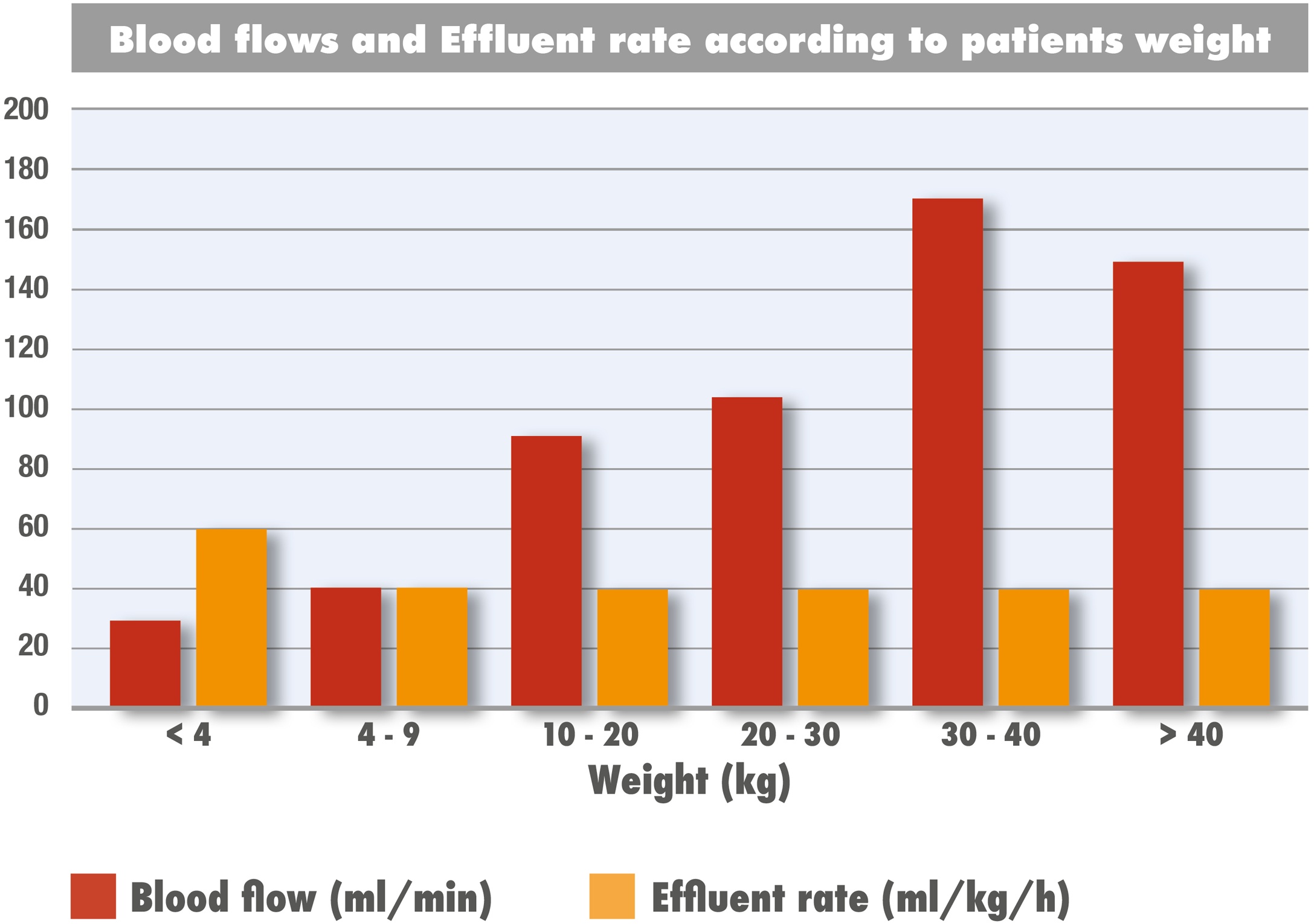
Histogram of the median blood flow rate and effluent rate according to weight categories in patients treated with hemoadsorption (HA) in combination with CRRT.

Fig. 2 presents the median blood flow and effluent doses according to weight categories. Overall median blood flow rate was 100 mL/min (IQR 60–148) and the median effluent dose was 40 mL/kg/hours (IQR 31; 40).

Packed Red Blood Cells (PRBCs) were added to the priming solution prior to the initiation of the extracorporeal circuit in 38 of 62 patients (61%), albumin in 19 of 62 patients (31%) and crystalloids in 4 of 62 cases (6%).

### Timing and clinical indications for treatment with CytoSorb®

In 48% of patients CytoSorb® treatment was initiated more than 48 h after PICU admission. In 27% of cases, therapy began within 24 h of admission, and in 26% of cases between 24 and 48 h.

**Fig. 3:**
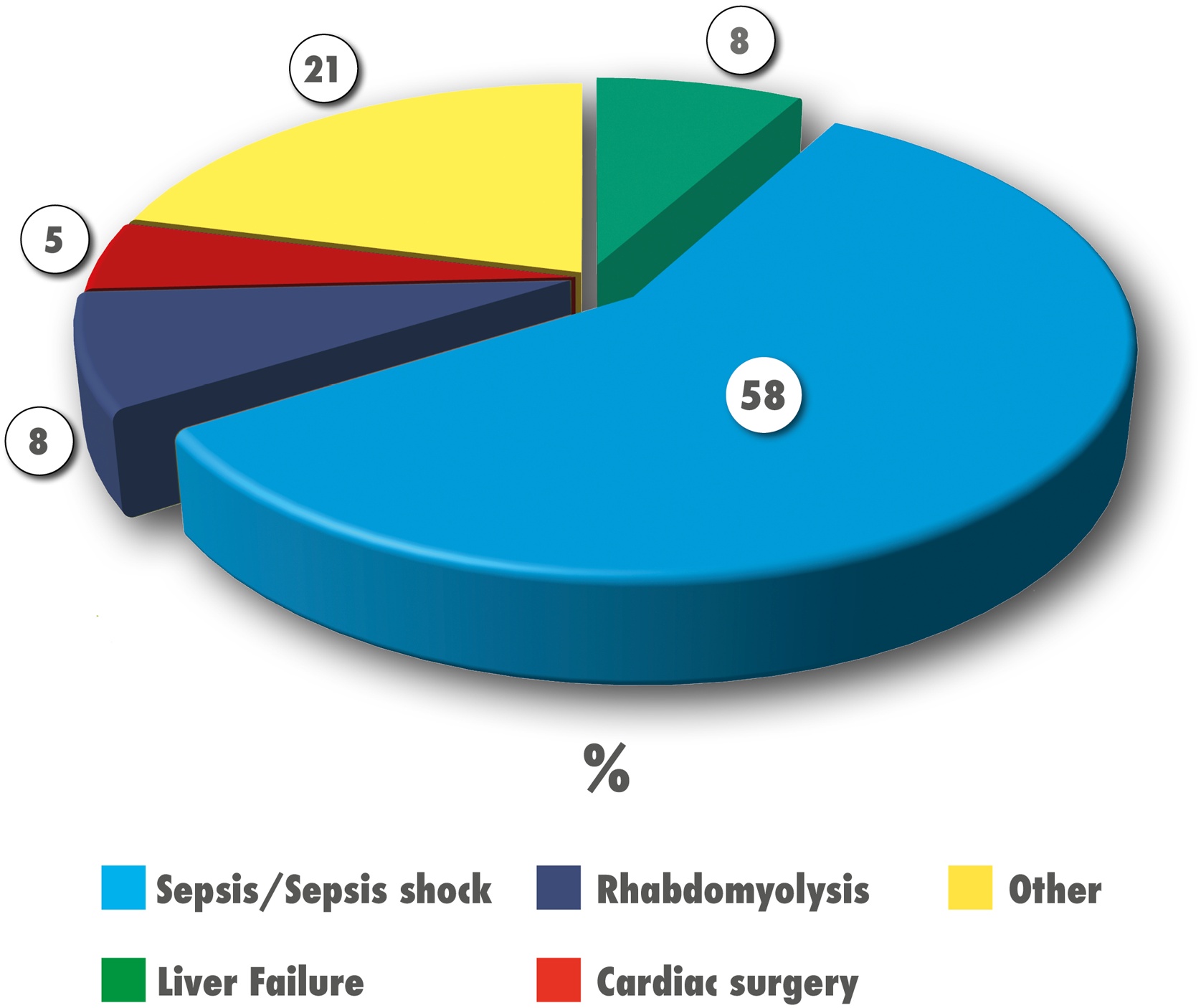
Pie-chart illustrating the percentage distribution of clinical indications of hemoadsorption (HA) with CytoSorb® in the study population.

Fig. 3 illustrates the distribution indications for CytoSorb® therapy. The most frequent indication was sepsis, accounting for 58% (36/62) of cases. Among these, 33 patients presented with septic shock, and 20 had catecholamine-refractory shock [10,11]. Multi-organ dysfunction syndrome (MODS) occurred in 89% of patients with sepsis. CytoSorb® was initiated within 24 h of septic shock onset (time of first vasoactive infusion meeting septic shock criteria) in 8 of 36 patients, and after 24 h in 28 of 36 patients. All patients treated with HA for septic shock also received CRRT.

Liver failure was the primary indication in 5/62 patients: the indications for treatment were hyper-bilirubinaemia (*n* = 2), acute liver failure (*n* = 2), and acute-on-chronic liver failure in (*n* = 1). All were on the liver transplant waiting list, and 2 had previously undergone liver transplant. The median total bilirubin level in this group was 22 mg/dL.

Severe rhabdomyolysis was the indication in 5 patients, caused by muscular ischemia (*n* = 2), viral infection (*n* = 1), metabolic disease (*n* = 1), and sickle cell crisis (*n* = 1). The median creatine kinase (CK) level was 127,870 U/L, and the median myoglobin level was 25,310 ng/mL.

CytoSorb® was applied in 3 patients during cardiopulmonary bypass CBP, all for infectious endocarditis.

In 21% of cases (12/62) the treatment was given for other indications: cytokine storm [hemophagocytic lymph histiocytosis (HLH) or cytokine release syndrome (CRS)] (*n* = 7), patients with haemolytic uremic syndrome (*n* = 2), acute respiratory distress syndrome (*n* = 1), drug intoxication (*n* = 1), and tumour lysis syndrome (*n* = 1).

### Adverse events

**Fig. 4:**
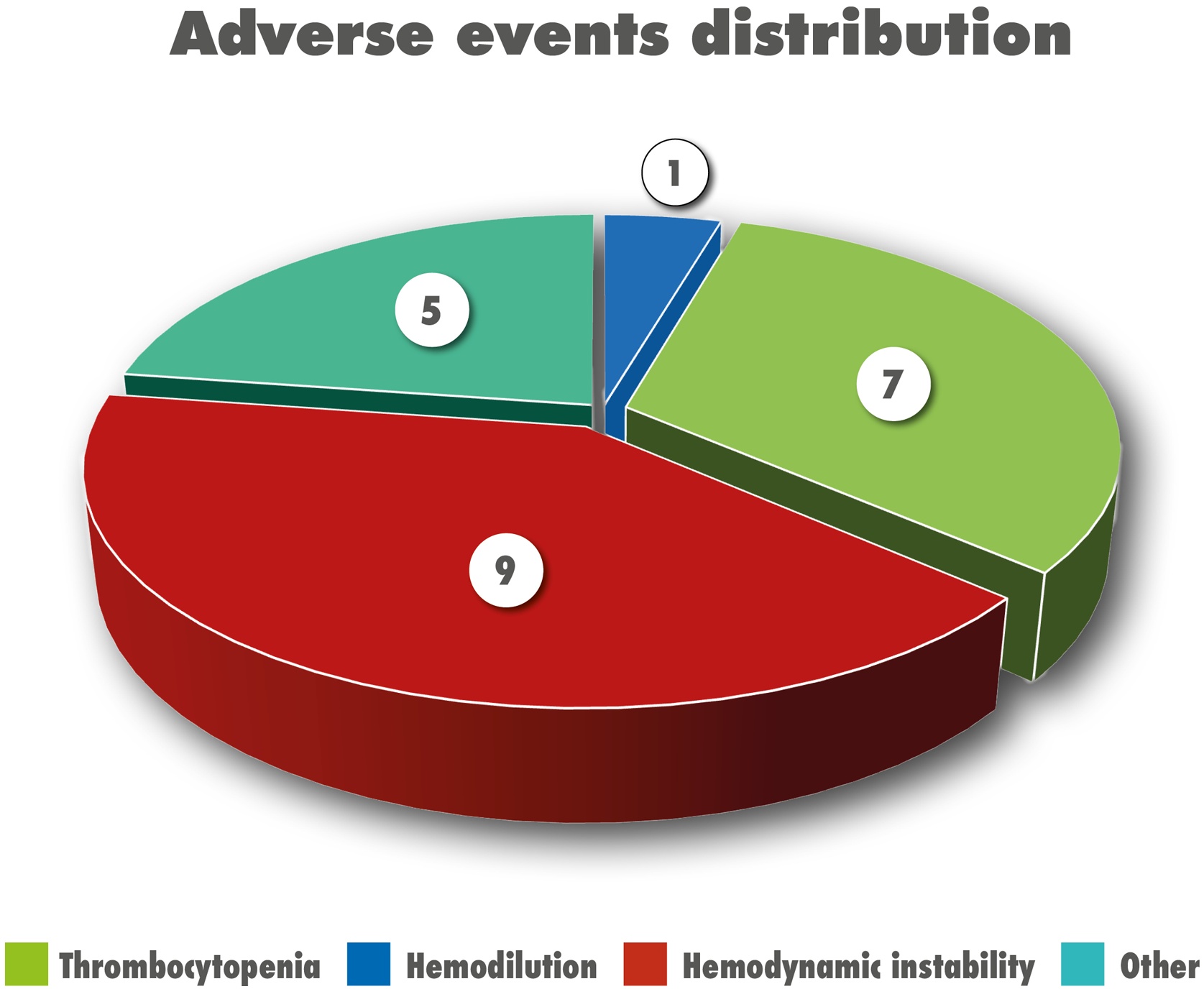
Pie-chart showing the distribution of types of adverse events during treatment with CytoSorb®.

Adverse occurred in 12 of 62 treated patients. Reported events included haemodynamic instability at the initiation of extracorporeal circulation (*n* = 9), thrombocytopaenia (*n* = 7), haemodilution (*n* = 1), and other events (*n* = 5) (Fig. 4), comprising coagulation abnormalities (*n* = 4), and circuit blockage (*n* = 1). Among patients experiencing complications, the median weight was 7.25 kg. Notably, 7 out of 9 patients who developed hemodynamic instability at the initiation of extracorporeal circulation received PRBCs in the priming solution

### Population survival

In the overall cohort, 28 of 62 patients (46%) died, with one patient lost to follow-up. The 28-day mortality was 33%, and the overall PICU mortality was 46%. At 28-days, mortality did not show a linear association with body weight: 53% (10/19) in patients weighing <14 kg, 6% (1/18) in those weighing 14–30 kg, and 37.5% (9/24) in those >30 kg. A logistic regression model of 28-day mortality was performed including six variables: body weight, PELOD-2 score, VIS score, haemato-oncological disease, other comorbidities, and use of advanced therapies (including immunotherapies with monoclonal antibodies and CAR-T cells). Results of the logistic regression analysis according to Firth’s penalized maximum estimation are presented in Table 2. After adjusting for other variables, children weighing <14 kg had 13-fold higher odds of 28-day mortality compared with those ≥14 kg. Patients with underlying haemato-oncologic disease had 5-fold higher odds of death, and those requiring advanced therapies had a 5-fold increased risk.

**Table 2:** Logistic regression analysis in the study population.

Firth’s penalized maximum likelihood estimation method and results:			
Risk factor	Odds ratio	95% CI	P-value
Weight (<14 kg vs. > = 14 kg)	13.04	(2.30, 73.8)	0.0037
Hemato-oncological disease (Yes vs. No)	4.86	(0.82, 28.82)	0.0820
Other comorbidities (Yes vs. No)	2.38	(0.58, 9.75)	0.2292
Any advanced therapy received (Yes vs. No)	5.27	(1.00, 27.79)	0.0500
PELOD-2 score (every 1 score increase)	0.91	(0.77, 1.08)	0.2752
VIS score (every 1 score increase)	1.00	(0.999, 1.003)	0.2702

Survival rates by indication were as follows: 100% in patients treated during CBP for endocarditis, 80% in rhabdomyolysis, 56% in septic shock, 54% in other indications, and 0% in liver failure.

## Discussion

Despite growing interest, HA has had limited application in the paediatric population, largely because most devices are designed for adults and due to technical challenges in implementing extracorporeal therapies in small children. These limitations have been highlighted in recent expert consensus statements, which identify them, together with the lack of high quality evidence, as key obstacles to the routine use of such techniques in paediatric critical care [12].

Our analysis has yielded several key findings. First, the patient population treated with HA had high clinical complexity and a substantial predicted mortality risk. a. The high prevalence of comorbidities further underscores their vulnerability to PICU-related complications. Importantly, 28-day mortality showed no linear association with body weight. Among the 61 evaluable patients, those weighing less than < 14 kg had a significantly higher risk of death. This finding is consistent with previous reports, including data from the paediatric continuous renal replacement therapy (pCRRT) registry, which have repeatedly demonstrated higher mortality in neonates and small infants supported with extracorporeal circuits [13,14].

Previous evidence [15,16] has shown that the use of CytoSorb® in small infants with blood flow rates <100 mL/min is feasible, reducing the risk of hypotension at the start of the extra-corporeal circuit. Moreover, circuit priming with PRBCs has been reported to prevent both hypotension and hemodilution at the initiation of the extracorporeal circuit [15,16]. Following our data, the analysis of blood flow rates by body weight demonstrated that, although designed for adults, CytoSorb® can be effectively used with flow rates as low as 30–40 mL/min. Furthermore, circuit priming with PRBCs was a common practice across centres and may help mitigate complications associated with extracorporeal therapies in smaller children.

Recently Borankulova and Sazanov [17] in a non-systematic review described the clinical outcomes in infants and neonates with sepsis treated with CytoSorb®. The authors reported the experience of 11 cases described by various authors, of which only 5 survived. This confirms the vulnerability of this category of small-size patients and also that the device is often applied late as a rescue therapy in cases with a very compromised clinical condition. However, the authors did not report the analysis of adverse events in their review [17].

A recent narrative review found HA to be generally safe and feasible in paediatric septic shock, with thrombocytopenia reported as the most common adverse event [18]. Our analysis of adverse events confirmed that hypotension at the initiation of the extracorporeal circuit and thrombocytopenia are the most frequently described adverse events, although uncommon (12 of 62 patients), and that in patients in whom events were recorded, the mean body weight was 7.5 kg, suggesting once again that lower body weight may be associated with an increased risk of complications during HA therapy. Minor adverse events, including circuit clotting, were infrequently observed. However, we acknowledge that due to the retrospective design of the study, some minor adverse events may have been underreported.

Patients with haematological and oncological diseases exhibited higher mortality rates. Our findings confirm previous reports linking these conditions to increased risk of death, which may influence long-term outcomes even after sepsis resolution [19]. In contrast, other comorbidities were not significantly associated with mortality. The combination of a high overall comorbidity burden and the presence of complex chronic conditions likely explains why PICU mortality exceeded 28-day mortality in our cohort.

Septic shock was the most common indication for CytoSorb® use, reflecting its perceived role as an adjuvant therapy for refractory sepsis and sepsis-associated multi-organ dysfunction (MODS) in Italian PICUs. Among children treated for septic shock, survival was 56%. Most of these patients, however, received treatment more than 24 h after septic shock onset, suggesting a possible window for earlier intervention to improve outcomes. In contrast, other indications — such as rhabdomyolysis and use during CPB surgery — were associated with excellent survival rates (88–100%). All children with liver failure, however, died, likely reflecting either refractoriness to treatment or initiation at a stage when the disease was already irreversible.

Our study has also some important limits. Firstly it is a retrospective observational study and what we observed and reported in terms of feasibility and safety cannot be claimed an effect of HA treatment in our population. Secondly the evidence is derived form a broader and less selected populations and some clinical indication such as liver failure and rhabdomyolysis are composed by very small groups of paediatric patients. Furthermore the small and unbalanced sample suggests a more cautious interpretation of the relationship between body weight and 28-day mortality. Finally within this analysis we did not investigate the impact of the HA on antibiotic removal. Indeed the potential removal of antimicrobials during HA remains an important consideration. Appropriate therapeutic drug monitoring, dose adjustment when indicated and close clinical monitoring are therefore recommended and should be included as outcomes in future studies.

## Conclusions

This multicentre retrospective study, the largest to date on paediatric CytoSorb® use, provide some insights into safety and feasibility of HA in critically ill children.

Most treatments were delivered in combination with CRRT, with septic shock as the leading indication. We observed a 28-day mortality of 33%, despite the cohort’s high severity of illness and comorbidity burden despite the cohort’s high severity scores and comorbidity burden. Children weighing <14 kg had significantly higher mortality and a greater risk of adverse events, highlighting the vulnerability of smaller patients to extracorporeal therapy–related complications. Hematologic–oncologic disease and the need for advanced therapies were also predictors of 28-day mortality. Our multicenter experience suggests that CytoSorb can be technically implemented in critically ill children, including those on CRRT/ECMO, with an acceptable safety profile in this context. The ongoing prospective CYTOPED registry will further inform optimal timing, dosing, and integration with other therapies, including antibiotics administration and other extracorporeal organ support techniques.

## CRediT authorship contribution statement

**GB:** Conceptualization, Data curation, Investigation, Methodology, Resources, Writing– original draft, Writing - review & editing. **SB, AT, MF, SG, SR, FP, EC, SS, PL**: Data curation, Investigation, Methodology, Writing - review & editing. **IG, AM, CC, GL, FC, AS, RS**: Methodology, Supervision, Validation, Writing - review & editing.

## Ethics approval and consent to participate

The studies involving humans were approved by Ethical Commitee Children Hospital Bambino Gesù and. The studies were conducted in accordance with the local legislation and institutional requirements. The participants provided their written informed consent to participate in this study.

## Funding

The author(s) declare financial support was received for the research, authorship, and/or publication of this article.

This work was supported also by the Italian Ministry of Health with “Current Research funds”.

The authors did not receive any specific funds for this manuscript.

## Consent for publication

The parents or child’s next of kin provided informed consent and all study procedures were conducted in accordance with the ethical standards and guidelines set forth by the REC for human experimentation and adhered to the principles of the Helsinki Declaration, 1975.

## Availability of data and materials

The raw data supporting the conclusions of this article will be made available by the authors, without undue reservation.

## Declaration of competing interest

The authors declare that the research was conducted in the absence of any commercial or financial relationships that could be construed as a potential conflict of interest.
